# Epigenetic reprogramming in autoimmune and immune-mediated skin disease

**DOI:** 10.3389/fimmu.2026.1915200

**Published:** 2026-08-26

**Authors:** Sophie M. Bilik, Garrett P. Kraft, Isabella Sharifi, Fiona M. Hanly-Jorda, Sarah Antonevich, Joaquin J. Jimenez, Takashi K. Satoh

**Affiliations:** 1 Dr. Phillip Frost Department of Dermatology and Cutaneous Surgery, University of Miami Miller School of Medicine, Miami, FL, United States; 2 University of Miami Miller School of Medicine, Miami, FL, United States; 3 Department of Biochemistry and Molecular Biology, University of Miami Miller School of Medicine, Miami, FL, United States; 4 Department of Dermatology and Allergy, University Hospital, Ludwig Maximilian University (LMU) of Munich, Munich, Germany

**Keywords:** autoimmune dermatology, chromatin remodeling, DNA methylation, epigenetics, histone modification, precision dermatology, skin-resident immune cells, tissue-resident memory T cells

## Abstract

**Background:**

Autoimmune skin diseases exhibit chronicity and site-specific relapse that are not fully explained by genetic susceptibility or cytokine signaling alone. Emerging evidence suggests that epigenetic reprogramming of skin-resident immune cells contributes to disease persistence and recurrence.

**Methods:**

We conducted a structured narrative review of the human literature by searching PubMed for studies published from database inception through March 1, 2026. The literature search was performed between February 1 and March 1, 2026 and focused on epigenetic mechanisms across major autoimmune dermatoses, including DNA methylation, histone modification, chromatin accessibility, non-coding RNA regulation, and emerging single-cell and spatial epigenomic technologies.

**Results:**

Current evidence suggests stable chromatin remodeling in keratinocytes and immune cells, including tissue-resident memory T cells, that persists after clinical resolution and may facilitate rapid disease reactivation. Environmental exposures further reinforce these epigenetic programs, contributing to disease heterogeneity, chronicity, and site-specific relapse.

**Conclusion:**

Collectively, these findings are consistent with a model in which autoimmune skin diseases are maintained by maladaptive epigenetic memory. Although causal inference remains limited by the relative scarcity of cell-type–resolved human epigenomic data and the predominance of cross-sectional studies, this framework suggests that achieving durable therapeutic remission may require reprogramming pathogenic chromatin states rather than solely suppressing inflammatory pathways.

## 1 Introduction

The skin is a highly specialized immunologic organ that integrates structural, cellular, and molecular defenses to maintain tissue homeostasis at the interface between the body and the external environment (1). This network comprises diverse skin-resident immune populations, including Langerhans cells, dendritic cells, tissue-resident T cells (TRM), innate lymphoid cells, macrophages, and mast cells, which function alongside keratinocytes and stromal cells to coordinate immune surveillance and response (1, 2). Unlike circulating immune cells, skin-resident populations exhibit long-term adaptation and persistence, enabling rapid local responses to recurrent stimuli (2, 3). While essential for protective immunity, dysregulation of these populations contributes to immune-mediated inflammatory and autoimmune skin diseases, including psoriasis, atopic dermatitis (AD), vitiligo, and cutaneous lupus erythematosus (CLE) (2–4). Epigenetic regulation has emerged as a key determinant of immune-cell identity, function, and memory. Mechanisms including DNA methylation, histone modification, chromatin accessibility, and non-coding RNA regulation enable immune cells to respond dynamically to environmental signals while maintaining lineage-specific transcriptional programs (5–7). In skin-resident immune populations, these processes support specialized phenotypes such as TRM persistence and rapid cell responses, while shaping dendritic-cell and macrophage activate states (5–9). Increasing evidence suggests that tissue residency, effector function, and inflammatory responsiveness are encoded through stable chromatin programs involving enhancer selection, chromatin accessibility, and transcription-factor occupancy (10–12). In this review, epigenetic memory refers to durable chromatin-associated regulatory states that persist after resolution of the initiating stimulus and influence subsequent cellular responses without altering the underlying DNA sequence (5–9). Such programs may involve DNA methylation, histone modifications, chromatin accessibility, higher-order chromatin organization, or non-coding RNA–mediated regulation that maintain altered transcriptional potential after apparent resolution of inflammation. Importantly, persistent epigenetic differences observed during active inflammation should not automatically be interpreted as evidence of epigenetic memory (5–7). Similar molecular signatures may instead reflect ongoing inflammatory signaling, altered cellular composition, sustained transcriptional activation, persistence of long-lived immune populations such as tissue-resident memory T cells, or repeated environmental stimulation (5–9). Throughout this review, we therefore reserve the term epigenetic memory for stable epigenetic programs that persist after resolution of the initiating stimulus and contribute to altered responses upon subsequent challenge or disease recurrence (5–12). For consistency, lesional skin refers to actively inflamed skin, non-lesional skin refers to clinically unaffected skin from affected individuals, peri-lesional skin refers to clinically unaffected skin immediately adjacent to a lesion, and clinically resolved skin refers to previously lesional skin following successful treatment or clinical remission.

Epigenetic reprogramming may provide a mechanistic framework for autoimmune disease persistence. Environmental and inflammatory stimuli may induce durable chromatin alterations that promote pathogenic immune states and sustain disease memory, contributing to site-specific recurrence, chronicity, and therapeutic resistance (13–17). These observations help explain features of autoimmune skin disease that are not fully accounted for by genetic susceptibility or cytokine signaling alone. Although current models have largely focused on genetic risk and inflammatory pathways (18–20), they do not fully explain disease heterogeneity, variability in treatment response, or relapse after apparent resolution. Epigenetic regulation integrates genetic predisposition, environmental exposure, and cellular memory, providing a unifying framework for understanding disease pathogenesis (21–26). In this review, we examine how epigenetic mechanisms shape skin-resident immune-cell identity and function, contribute to autoimmune disease pathogenesis, and establish durable inflammatory memory. We further discuss disease-specific epigenetic signatures, environmental drivers of epigenetic change, emerging technologies for mapping the cutaneous epigenome, and therapeutic strategies aimed at reprogramming pathogenic chromatin states.

## 2 Methods

### 2.1 Literature search strategy

A structured literature search was conducted in PubMed to identify studies examining epigenetic regulation of skin-resident immune cells in autoimmune and inflammatory skin disease. The literature search was conducted between February 1 and March 1, 2026, and included publications from database inception through March 1, 2026. Search terms combined Medical Subject Headings (MeSH) and free-text keywords related to epigenetics, chromatin regulation, immune cell populations, skin biology, and inflammatory skin diseases using Boolean operators. A representative search strategy included:

(“Epigenesis, Genetic” OR epigenetic regulation OR DNA methylation OR histone modification OR chromatin accessibility OR ATAC-seq) AND (skin OR cutaneous OR skin immunity) AND (“T-Lymphocytes” OR tissue-resident memory T cells OR dendritic cells OR macrophages OR mast cells OR innate lymphoid cells) AND (psoriasis OR atopic dermatitis OR vitiligo OR cutaneous lupus OR inflammatory skin disease”

The search strategy was refined iteratively to maximize sensitivity and relevance. Additional publications were identified through manual screening of reference lists from selected articles and landmark reviews. The complete search methodology is summarized in **Table 1**.

**Table 1:** Summary of literature search strategy for narrative review on epigenetic regulation in cutaneous immunity.

Category	Description
Review Type	Structured narrative literature review
Primary Database	PubMed
Search Period	February 1–March 1, 2026
Publication Date Range	Database inception through March 1, 2026
Representative Search Strategy	(“Epigenesis, Genetic” OR epigenetic regulation OR DNA methylation OR histone modification OR chromatin accessibility OR ATAC-seq) AND (skin OR cutaneous OR skin immunity) AND (“T-Lymphocytes” OR tissue-resident memory T cells OR dendritic cells OR macrophages OR mast cells OR innate lymphoid cells) AND (psoriasis OR atopic dermatitis OR vitiligo OR cutaneous lupus OR inflammatory skin disease)
MeSH Terms	*Epigenesis, Genetic*; *DNA Methylation*; *Histone Modification*; *Chromatin Accessibility*; *T-Lymphocytes*; *Dendritic Cells*; *Macrophages*; *Mast Cells*; *Skin*; *Autoimmune Diseases*; *Psoriasis*; *Dermatitis, Atopic*; *Lupus Erythematosus, Cutaneous*
Keywords	Epigenetic regulation; chromatin accessibility; ATAC-seq; histone acetylation; DNA methylation; tissue-resident memory T cells (TRM); skin immunity; dendritic cells; macrophages; mast cells; innate lymphoid cells; psoriasis; vitiligo; cutaneous lupus; immune memory; disease recurrence; inflammatory skin disease
Language Restrictions	English-language publications
Study Types Included	Peer-reviewed primary research articles, translational studies, clinical investigations, and high-quality review articles
Study Selection Process	Titles and abstracts were screened for relevance, followed by full-text review of potentially eligible articles. Reference lists of selected articles and landmark reviews were manually screened to identify additional relevant studies.
Number of Reviewers	Two reviewers
Records Identified	Approximately 650 records retrieved through database searching and manual reference screening.
Publications Included	200
Inclusion Criteria	Peer-reviewed studies examining epigenetic mechanisms in immune cells; studies involving skin or skin-resident immune populations; human experimental, translational, or clinical studies; selected animal and *in vitro* studies providing mechanistic insight relevant to cutaneous immunity; studies linking epigenetic regulation to immune function or disease pathogenesis.
Exclusion Criteria	Studies lacking direct relevance to cutaneous immunity; articles without epigenetic or chromatin-level analyses; publications not addressing immune cell regulation within the context of skin biology.
Additional Sources	Manual screening of reference lists from selected articles and landmark reviews
Data Extraction	Immune cell type, epigenetic mechanism, disease context, experimental methodology, and principal functional outcomes
Synthesis Method	Qualitative narrative synthesis organized into four thematic categories because of methodological heterogeneity across included studies

### 2.2 Study selection and data synthesis

Eligible studies included peer-reviewed primary research and review articles examining epigenetic mechanisms in immune cells with direct relevance to skin biology or cutaneous immune populations. Preference was given to human studies incorporating molecular or genomic approaches, including DNA methylation profiling, chromatin accessibility analyses, histone modification studies, and single-cell sequencing. Studies lacking direct relevance to cutaneous immunity or epigenetic regulation were excluded. The literature search, screening, and study selection were conducted by two reviewers. Titles and abstracts were initially screened for relevance, followed by full-text evaluation of potentially eligible articles. A total of 200 publications were ultimately included in the final review. Data extracted included immune cell type, epigenetic mechanism, disease context, and functional outcomes. Findings were synthesized into four thematic categories (1): epigenetic regulation of tissue residency and immune memory (2), chromatin remodeling in inflammatory skin disease (3), cell-type–specific epigenetic programs, and (4) disease-associated epigenetic reprogramming and recurrence. This review was conducted as a structured narrative review aimed at providing a comprehensive synthesis of current evidence rather than a formal systematic review. Accordingly, PRISMA reporting guidelines were not applied.

## 3 Skin-resident immune cells

Human single-cell and spatial transcriptomic studies show that skin is organized into anatomically distinct epidermal, dermal, adnexal, and perivascular cellular neighborhoods in which immune cells interact closely with keratinocytes, fibroblasts, endothelial cells, and other stromal elements to maintain barrier function and immune surveillance (27). In psoriasis, single-cell analyses of clinically resolved skin following IL-23 blockade demonstrate that clinically resolved skin can retain residual inflammatory stromal and keratinocyte programs after visible resolution, supporting the concept of localized inflammatory memory rather than complete tissue reset (16, 28). Among the resident immune populations implicated in these processes, tissue-resident memory T cells (TRM), dendritic cells, macrophages, innate lymphoid cells (ILC), and mast cells play central roles in coordinating local immune responses. Increasing evidence suggests that their functional specialization and persistence are shaped by tissue-specific transcriptional and epigenetic programs, making them plausible candidates for mediating disease chronicity and recurrence.

### 3.1 Tissue-resident memory T-cells

Human skin contains abundant TRM cells, typically identified by CD69, with or without CD103, that occupy epidermal and dermal niches and display phenotypic and functional features distinct from circulating memory T cells (29–31). It is more accurate, however, to describe these cells as predominantly tissue-retained rather than permanent, because a fraction of human cutaneous CD4+CD103+ TRM can downregulate CD69, re-enter circulation, and reseed skin (30). Ex vivo human skin TRM also retain functional cytokine and cytotoxic potential, supporting their role as a poised local memory compartment (31, 32). In psoriasis, paired analyses of clinically resolved lesions contain psoriasis-specific IL-17-producing αβ T-cell clones, and lesional CD8+CD103+ TRM are enriched for IL-17A production, supporting a role for persistent lesional TRM in site-specific relapse (15, 33). Human ATAC-seq studies further show widespread increased chromatin accessibility in psoriatic skin, and analysis of clinically resolved skin obtained after treatment demonstrates residual accessibility at inflammatory loci including S100A7, S100A8, S100A9, and IL36G, consistent with epigenetic poising after apparent resolution (11, 16). In vitiligo, lesional epidermis contains resident-like cytotoxic CD8+ T cells with TRM-associated markers including CD49a, and these cells can persist during systemic corticosteroid therapy (34, 35). Taken together, currently available human data suggest persistent lesional resident T-cell populations within clinically resolved psoriasis and vitiligo, but direct patient-level epigenomic proof at TRM-specific residency and tissue-egress loci remains limited and should not be overstated.

### 3.2 Langerhans cell and dermal-dendritic cells

Human epidermal Langerhans cells are antigen-presenting cells identified by CD1a and langerin/CD207 (36, 37). In human AD and allergic contact dermatitis, lesion-based studies show altered LC activation states and trafficking, supporting a context-dependent role in inflammation (38–40). Direct human chromatin-level LC data in inflammatory dermatoses remain limited. Accordingly, it is more accurate to say that transcriptomic studies show disease-associated LC reprogramming rather than a fully defined stable enhancer architecture in patients (41). The human dermis also contains phenotypically distinct dendritic-cell subsets, including CD1c+ and CD141+ populations (42). In psoriasis, single-cell profiling identified CD14+ DC3 cells in lesional skin that co-produce IL1B and IL23A, supporting a role for dermal myeloid dendritic cells in the IL-23/Th17 axis (43). In CLE, lupus-enriched CD16+ dendritic cells acquire interferon-educated pro-inflammatory phenotypes within patient skin (44).

### 3.3 Macrophages and innate lymphoid cells

Macrophages and ILCs are resident innate immune populations that contribute to tissue homeostasis, barrier defense, and local inflammatory responses through continuous interactions with epithelial, stromal, and adaptive immune cells (45, 46). Human skin single-cell studies identify macrophage populations within normal and diseased dermis, and in CLE integrated single-cell and spatial transcriptomics show an interferon-rich skin environment with marked myeloid-cell reprogramming (27, 44). However, direct human evidence that specific histone-acetylation or DNA-methylation programs lock cutaneous lupus macrophages into stable pathogenic states remains sparse, so that mechanistic language should be treated as unproven in patient tissue. For innate lymphoid cells, the strongest human skin evidence currently supports two disease settings. In psoriasis, NKp44+ ILC3 are increased in skin and blood, their circulating frequency correlates with disease severity, and they decline after anti-TNF therapy (47). In AD, lesional skin contains activated ILC populations, and IL-13 blockade reduces inducible T-cell costimulatory-positive ILCs in lesions (48). Direct human skin epigenetic studies of ILC polarization remain limited; the better-supported wording is therefore transcriptional plasticity in response to the local cytokine milieu, not a settled clinical epigenetic mechanism.

### 3.4 Mast cells

Human skin mast cells are FcϵRI- (high-affinity immunoglobulin E receptor) and KIT/CD117-positive effector cells positioned near superficial dermal vessels and nerves, which fits their central role in wheal formation and sensory symptoms (49, 50). In AD, mast cells are found in close association with peripheral nerves in lesional skin, supporting a neuroimmune contribution to itch and inflammation (51). Beyond their established role in allergic inflammation and pruritus, mast cells may also contribute to local immune memory through interactions with resident T cells, sensory neurons, and stromal cells, although the epigenetic mechanisms regulating these functions remain incompletely defined (52).

## 4 Epigenetic mechanisms regulating skin immune cells

### 4.1 DNA methylation

DNA methylation is a relatively stable yet reversible epigenetic mechanism that regulates gene expression without altering DNA sequence (53, 54). In mammals, methylation is established by DNA methyltransferases (DNMT3A and DNMT3B), maintained during cell division primarily by DNMT1, and actively reversed through ten-eleven translocation (TET) enzyme–mediated demethylation, enabling durable yet adaptable regulation of transcriptional activity (55, 56).

Within the skin, DNA methylation helps preserve long-lived transcriptional programs in both epithelial and immune cells that are continuously exposed to environmental and inflammatory stimuli. By maintaining lineage-specific gene expression while limiting inappropriate activation of alternative programs, DNA methylation contributes to epidermal homeostasis and immune-cell identity (54, 57). DNMT1 is enriched in undifferentiated epidermal progenitor cells, and its loss leads to impaired proliferation and premature differentiation, underscoring the role of methylation in maintaining the epithelial niche that shapes local immune function (57). Similarly, altered methylation landscapes in CD4^+^ T cells have been associated with changes in cytokine expression and functional programming that may influence the persistence of tissue-adapted and memory-like immune populations (58). In immune-mediated and autoimmune skin diseases, DNA methylation is particularly relevant because it provides one mechanism through which repeated environmental and inflammatory exposures may become stably encoded within epithelial and immune cells. Rather than functioning as a static regulatory mark, methylation may contribute to persistent transcriptional programs that are consistent with long-lived inflammatory memory and recurrent disease activity (54, 59). Genome-wide methylation profiling, most commonly performed using bisulfite-based sequencing and related methylome approaches, enables high-resolution characterization of DNA methylation patterns in skin and immune cells (60–62). These technologies have been instrumental in identifying disease-associated methylation signatures and will likely continue to improve understanding of how persistent epigenetic programs contribute to immune-cell function, tissue memory, and disease recurrence.

### 4.2 Histone modifications: acetylation and methylation

Histone modifications, particularly acetylation and methylation, are key regulators of chromatin accessibility and transcriptional control in skin-resident immune cells. By altering how tightly DNA is packaged around histone proteins, these modifications regulate the accessibility of promoters and enhancers to transcription factors and thereby influence gene expression (63). Histone acetylation, mediated by histone acetyltransferases (HATs), is generally associated with open chromatin and active transcription, whereas histone deacetylases (HDACs) promote chromatin compaction and transcriptional repression. Histone methylation is more context dependent and may either activate or repress transcription depending on the modified residue. For example, H3K4me3 and H3K27ac are characteristic of active promoters and enhancers, whereas H3K27me3 is associated with transcriptionally repressed chromatin and maintenance of lineage-specific gene silencing (64–66). Within the skin, histone modifications help balance rapid responsiveness to environmental stimuli with preservation of long-term cellular identity. Dynamic histone acetylation enables rapid transcriptional responses to cytokines, microbial products, and tissue injury, whereas histone methylation contributes to more stable maintenance of transcriptional programs. Together, these complementary mechanisms may allow repeated inflammatory exposures to be translated into relatively persistent chromatin configurations capable of influencing subsequent immune responses.

In immune-mediated inflammatory and autoimmune skin diseases, dysregulated histone modifications have been associated with persistent activation of inflammatory pathways, altered epithelial differentiation, and impaired barrier function (67, 68). Although direct evidence linking specific histone modifications to long-term immunological memory in human skin remains limited, persistent patterns of histone acetylation and methylation are consistent with chromatin-based mechanisms that may contribute to durable inflammatory programs and disease recurrence. Consequently, histone-modifying enzymes, including HDACs and components of the Polycomb complexes, have emerged as promising therapeutic targets aimed at restoring appropriate transcriptional regulation in chronic inflammatory skin disease.

### 4.3 Chromatin accessibility and remodeling

Chromatin accessibility reflects whether regulatory DNA is physically available to transcription factors and the transcriptional machinery, providing a functional readout of which promoters and enhancers are poised for activation (67). Unlike DNA methylation and histone modifications, which mark regulatory potential, chromatin accessibility captures the transcriptional readiness of genomic regions at a given time point. This makes it particularly informative in skin-resident immune cells, where repeated environmental and inflammatory exposures require rapid but coordinated transcriptional responses.

Accessibility landscapes are established early during immune cell differentiation and are further refined upon tissue entry. In CD8^+^ T cells, precursors of TRM populations already exhibit distinct chromatin accessibility profiles compared with circulating counterparts, particularly at genes governing tissue retention, residency, and recirculation (68). Once established, TRM cells across different tissues share a conserved core accessibility program while retaining tissue-specific accessible regions, indicating that local environmental cues imprint durable, site-specific regulatory states (69). In the skin, this is especially relevant given the central role of TRM cells in immune surveillance and recurrent inflammatory responses.

Chromatin remodeling complexes and lineage-defining transcription factors cooperate to establish and maintain these accessibility landscapes (64, 70). For example, the transcription factor Runx3 exemplifies this principle: it promotes tissue residency by maintaining accessibility at residency-associated loci while repressing genes involved in tissue egress and has been shown to be required for TRM differentiation across multiple non-lymphoid tissues (68). Tissue-derived signals further shape these programs, as fibroblastic reticular cells can remodel chromatin accessibility in activated CD8^+^ T cells to promote survival, metabolic adaptation, and TRM differentiation, illustrating how stromal–immune interactions may reinforce long-term chromatin states (71).

Persistent alterations in chromatin accessibility have therefore emerged as one of the strongest candidate mechanisms underlying epigenetic memory in skin-resident immune cells. By maintaining regulatory elements in accessible configurations, repeated inflammatory exposure may prime immune and epithelial cells for enhanced responses upon subsequent stimulation, potentially contributing to disease recurrence and chronicity. However, direct evidence linking these persistent accessibility states to long-term clinical relapse in human inflammatory skin disease remains limited and warrants further investigation.

### 4.4 Non-coding RNAs

Non-coding RNAs provide an additional regulatory layer that modulates gene expression through both post-transcriptional and chromatin-associated mechanisms. Importantly, not all non-coding RNA dysregulation constitutes epigenetic memory. While some long non-coding RNAs directly influence chromatin organization and transcriptional regulation, many microRNAs primarily exert post-transcriptional effects on mRNA stability and translation. Accordingly, non-coding RNA dysregulation should only be interpreted as evidence of epigenetic memory when linked to persistent chromatin-associated regulatory programs. Unlike DNA methylation and histone modifications, which primarily regulate transcriptional potential, non-coding RNAs fine-tune how epithelial and immune cells respond to inflammatory signals, often acting within interconnected regulatory networks.

MicroRNAs (miRNAs) are the most extensively studied non-coding RNAs in immune-mediated inflammatory and autoimmune skin diseases. In psoriasis, miR-31 is markedly overexpressed in keratinocytes, where it enhances NF-κB signaling and promotes production of pro-inflammatory cytokines and chemokines while contributing to epidermal hyperplasia (69, 70). In vitiligo, miR-155 and miR-21 have been implicated in regulating the balance between regulatory and effector T-cell populations, suggesting that non-coding RNAs may influence both cytotoxic activity and immune tolerance (71).

Long non-coding RNAs (lncRNAs) are increasingly recognized as regulators of epidermal differentiation and inflammatory signaling. Transcriptomic studies demonstrate widespread lncRNA dysregulation in psoriatic skin, and functional studies have identified several lncRNAs, including MEG3, H19, and CYDAER, that influence keratinocyte proliferation, differentiation, and inflammatory signaling through interactions with microRNAs and downstream signaling pathways (72–75). These observations suggest that lncRNAs contribute to coordinated regulation of epithelial and immune responses rather than functioning as isolated molecular effectors.

An emerging concept is that non-coding RNAs operate within integrated regulatory networks. Many lncRNAs function as competing endogenous RNAs (ceRNAs), modulating the availability of microRNAs and thereby influencing expression of shared target genes. In psoriasis, transcriptome-wide analyses have identified lncRNA-associated ceRNA networks linked to interferon-γ and JAK–STAT signaling, supporting the concept that multiple RNA species converge on common inflammatory pathways (76).

Although non-coding RNAs generally represent more dynamic regulatory mechanisms than DNA methylation or chromatin accessibility, accumulating evidence suggests that they cooperate with chromatin-based processes to reinforce persistent inflammatory programs within epithelial and immune cells. Their ability to coordinate multiple signaling pathways simultaneously also makes them attractive candidates for therapeutic modulation of disease-associated immune memory, although their precise contribution to long-term clinical recurrence remains incompletely understood.

### 4.5 Three-dimensional chromatin architecture

Three-dimensional (3D) chromatin architecture refers to the spatial organization of the genome within the nucleus, which brings genomic regions that are distant in linear sequence into physical proximity. This higher-order structure may facilitate interactions between promoters, enhancers, insulators, and other regulatory elements, thereby influencing gene expression without altering DNA sequence (77, 78). Genome-wide chromosome conformation capture studies have shown that chromatin is organized into hierarchical structures, including compartments, topologically associating domains (TADs), and loops, that are non-random and closely linked to transcriptional regulation (77, 78). Chromatin loops, often anchored by CCCTC-binding factor (CTCF; an architectural protein that organizes chromatin structure) and cohesin, frequently connect enhancers to their target promoters, providing a structural framework for long-range gene regulation.

In skin biology, 3D genome organization is particularly important because many regulatory elements do not act on the nearest gene. Instead, chromatin looping enables cell-type-specific interactions between distal regulatory regions and target promoters, which may shape gene expression programs in keratinocytes and skin-resident immune cells. This is highly relevant in inflammatory skin diseases such as psoriasis, where disease-associated variants are enriched in non-coding regions. H3K27ac HiChIP (a technique combining chromatin interaction mapping with enhancer-associated histone marks) in keratinocytes and skin-derived CD8^+^ T cells has demonstrated that chromatin interactions can identify biologically relevant target genes for dermatologic traits, often diverging from nearest-gene assumptions (79). Similarly, Capture Hi-C studies show that psoriasis-associated loci form long-range interactions with distal genes such as KLF4 in a cell-type-specific manner, with enhancer activity enriched in keratinocytes (79).

This framework is especially important for interpreting genetic risk in autoimmune skin disease. Many risk variants identified through genome-wide association studies reside in non-coding regions, where their functional effects are not immediately apparent. Three-dimensional chromatin mapping can help link these variants to their true target genes, often located hundreds of kilobases away. In psoriasis, such approaches have reassigned disease-associated loci to distal regulatory targets, demonstrating that enhancer–promoter interactions, rather than genomic proximity, may influence gene regulation in disease-relevant cell types (78, 79). More broadly, integrated single-cell chromatin and transcriptomic analyses have shown that cell-type-specific enhancer–gene maps can be used to prioritize relevant genes, pathways, and causal variants in skin and hair disorders, revealing regulatory relationships that would be missed by bulk or proximity-based approaches alone (80). Collectively, these findings support the concept that 3D genome architecture represents an important layer connecting genetic variation to epigenetic regulation and disease pathogenesis in the skin.

**Figure 1:**
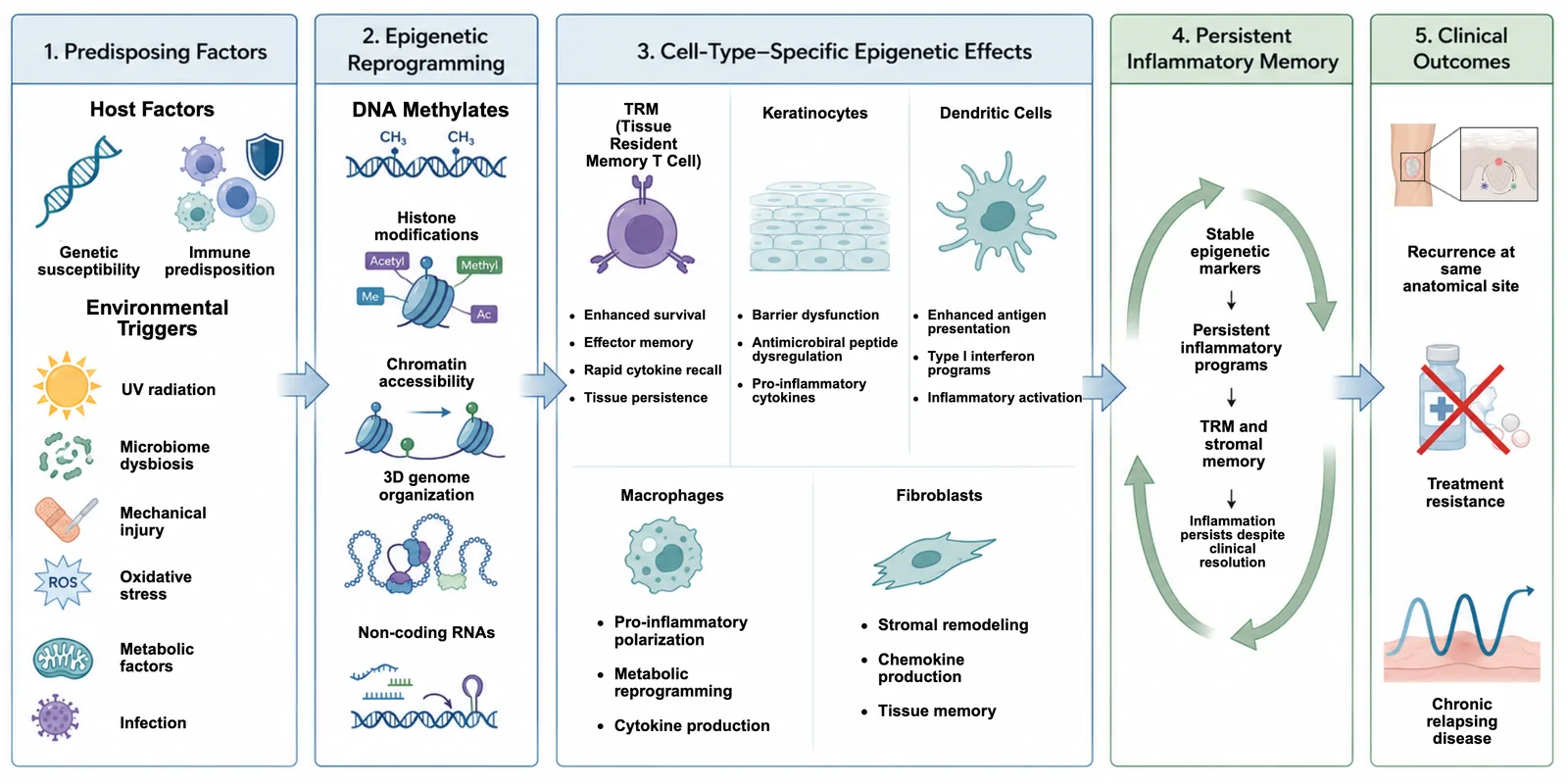
Proposed model of maladaptive epigenetic memory underlying persistence and recurrence in autoimmune skin disease. Genetic susceptibility and environmental exposures, including ultraviolet radiation, microbial dysbiosis, mechanical injury, oxidative stress, metabolic factors, and infection, promote epigenetic reprogramming through DNA methylation, histone modifications, chromatin accessibility, three-dimensional genome organization, and non-coding RNA regulation. These alterations establish cell-type–specific programs in tissue-resident memory T (TRM) cells, keratinocytes, dendritic cells, macrophages, and fibroblasts that reinforce persistent inflammatory memory despite clinical resolution. The resulting epigenetic programs may contribute to site-specific recurrence, treatment resistance, and chronic relapsing disease while highlighting opportunities for biomarker development and targeted epigenetic therapies.

The key molecular components underlying these processes and their relevance to skin disease are summarized in **Table 2**, which highlights major epigenetic mechanisms, associated enzymes and regulatory factors, and their functional contributions to cutaneous immune homeostasis and pathology. **Figure 1** provides an overview of the proposed model through which genetic susceptibility and environmental exposures induce cell-specific epigenetic reprogramming that contributes to persistent inflammatory memory, disease recurrence, and potential therapeutic opportunities.

**Table 2:** Epigenetic mechanisms in skin immune cells: enzymes and disease relevance.

Mechanism	Key enzymes/readers	Disease relevance in skin	References
DNA Methylation	DNA methyltransferase 1, DNA methyltransferase 3 alpha, DNA methyltransferase 3 beta, ten-eleven translocation enzymes, ubiquitin-like with PHD and RING finger domains 1	Regulates stable transcriptional memory, lineage identity, and inflammatory responsiveness in epidermal and immune compartments; altered methylation contributes to persistent pathogenic programs in autoimmune skin disease	(53–62)
Histone Acetylation	Histone acetyltransferases including p300/CREB-binding protein; histone deacetylases; bromodomain readers	Controls enhancer activation and rapid inflammatory gene expression; relevant to keratinocyte activation, dendritic cell maturation, and T-cell inflammatory signaling in psoriasis-like inflammation	(63–68, 188–190)
Histone Methylation	Enhancer of zeste homolog 2, polycomb repressive complex 2, jumonji domain containing 3, ubiquitously transcribed tetratricopeptide repeat gene X	Maintains repressive or permissive chromatin states that influence keratinocyte proliferation, differentiation, and immune-cell programming; enhancer of zeste homolog 2/histone H3 lysine 27 trimethylation is increased in psoriatic epidermis	(64–67, 192)
Chromatin Remodeling	ATP-dependent remodeling complexes; transcription factors linked to remodeling such as Runx3	Determines accessibility of regulatory DNA and stabilizes tissue-resident immune cell states; relevant to tissue-resident memory T-cell persistence and recall responses in skin	(10–12, 68–71)
Non-coding RNA	MicroRNAs, long non-coding RNAs, competing endogenous RNA networks	Modulates inflammatory signaling, keratinocyte proliferation, differentiation, and immune-cell communication; dysregulated microRNA and long non-coding RNA networks are implicated in psoriasis and other inflammatory skin states	(69–76)
3D Chromatin Architecture	CCCTC-binding factor, cohesin-associated looping machinery, enhancer-promoter interaction networks	Links distal regulatory elements to target genes in keratinocytes and skin immune cells; helps explain how non-coding risk loci drive pathogenic gene expression in skin disease	(77–80)

## 5 Epigenetic memory in immune-mediated and autoimmune diseases

The following subsections examine selected dermatologic conditions through the lens of human epigenetic research. For each condition, the discussion is organized around four core themes: disease background and epidemiology, which situates the condition within the broader landscape of dermatologic disease burden; key epigenetic findings, which synthesize insights from human studies of DNA methylation, histone modification, and noncoding RNA; mechanistic interpretation, which integrates these findings into models of how chromatin dynamics influence disease-relevant immune and epithelial processes; and therapeutic relevance, which highlights potential implications for biomarker development, patient stratification, and future epigenetic interventions.

To facilitate interpretation of the literature, the current evidence supporting epigenetic mechanisms across the major autoimmune skin diseases is summarized in **Table 3**, which classifies the principal studies according to disease, cell or tissue studied, epigenetic methodology, evidence source, study design, principal findings, and key limitations.

**Table 3:** Summary of the current evidence supporting epigenetic mechanisms in autoimmune skin diseases.

Disease	Cell/tissue studied	Epigenetic method(s)	Evidence source	Study design	Principal findings	Major limitations	Key references
Psoriasis	Lesional skin, keratinocytes, CD4^+^/CD8^+^ T cells, TRM	DNA methylation, ATAC-seq, H3K27ac profiling, scRNA-seq	Predominantly human (lesional skin, sorted T cells); complementary animal and mechanistic studies	Human case-control, single-cell, longitudinal treatment studies	Persistent chromatin accessibility, disease-associated methylation, residual inflammatory memory following clinical resolution	Limited longitudinal epigenomic studies; incomplete causal validation; relatively few functional perturbation studies	(11, 16, 17, 58, 59, 84–88)
Atopic Dermatitis	Epidermis, keratinocytes, CLA^+^ memory T cells, monocytes	DNA methylation, miRNA profiling, transcriptomics	Predominantly human (lesional skin and peripheral immune cells); limited mechanistic *in vitro* studies	Human case-control and translational studies	Tissue-specific methylation, TSLP and FCER1G promoter demethylation, skin-homing T-cell epigenetic signatures	Predominantly cross-sectional studies; limited chromatin accessibility and histone modification data; few longitudinal datasets	(90–94)
Cutaneous Lupus Erythematosus	Lesional skin, keratinocytes, naïve CD4^+^ T cells	DNA methylation, miRNA profiling	Predominantly human (lesional skin and peripheral blood); limited mechanistic studies	Human observational and translational studies	Epigenetic regulation of interferon signaling, antigen presentation, and UV-induced photosensitivity	Limited cell-resolved profiling; reliance on peripheral blood; few longitudinal studies	(96–99)
Vitiligo	Lesional/peri-lesional skin, CD8^+^ T cells, PBMCs	DNA methylation, miRNA profiling, genome-wide methylome analysis	Predominantly human (skin and peripheral immune cells); complementary mechanistic studies	Human observational and translational studies	Epigenetically primed cytotoxic CD8^+^ T-cell responses; peri-lesional methylation changes preceding depigmentation	Limited longitudinal studies; limited TRM-specific chromatin profiling; limited functional validation	(103–107)
Alopecia Areata	Scalp tissue, PBMCs, peripheral immune cells	scATAC-seq, single-cell multiome, DNA methylation, histone acetylation	Predominantly human (scalp tissue and peripheral immune cells)	Human PBMC studies and single-cell multiomic analyses	Progressive chromatin remodeling in cytotoxic lymphocytes and innate immune cells correlates with disease severity	Small cohorts; limited longitudinal studies; limited evidence for persistent resident immune memory	(81, 110–112)

These shared and disease-specific features are summarized in **Table 4**, which consolidates the dominant cell types, characteristic epigenetic signatures, functional consequences, and potential therapeutic implications across psoriasis, AD, CLE, vitiligo, and alopecia areata (AA).

**Table 4:** Disease-specific epigenetic signatures in cutaneous autoimmune conditions.

Disease	Key cell types	Epigenetic signature	Functional consequence	Potential therapy
Psoriasis	Lesional keratinocytes; CD4+ T cells; CD8+ T cells	Lesion-wide CpG dysmethylation enriched in PSORS loci, H3K27ac gain at overexpressed genes, and disease-discriminating T-cell methylation programs (58, 59, 83–87)	Stabilization of inflammatory transcription, epidermal hyperproliferation, and durable pathogenic T-cell programs	Methylation-based stratification and pharmacodynamic monitoring of cytokine blockade
Atopic Dermatitis	Lesional keratinocytes; monocytes; skin-homing CD4+ CLA+ memory T cells	Epidermis-biased methylome remodeling, TSLP promoter demethylation, FCER1G demethylation, IL13 hypomethylation, and miRNA rewiring in CLA+ T cells (89–93)	Persistent epithelial alarmin release, amplified FcϵRI signaling, and epigenetically reinforced skin-homing type 2 memory responses	Biomarker development for alarmin/type 2 blockade and future miRNA-guided monitoring
Cutaneous Lupus	Lesional/peri-lesional skin; naïve CD4+ T cells; circulating miRNA compartment	miR-31/miR-485-3p dysregulation in DLE, miR-885-5p loss with NF-κB activation, rash-specific CD4 methylation at antigen-presentation pathways, and Hippo-pathway hypomethylation in lupus keratinocytes (95–98)	Photosensitive apoptosis, increased immune recruitment, and enhanced antigen-handling capacity	miRNA- and pathway-prioritization strategies focused on NF-κB, antigen presentation, and photosensitivity biology
Vitiligo	CD8+ T cells; CD4+ T cells/Tregs; peri-lesional keratinocytes and melanocytes	PBMC methylation abnormalities, PRF1 promoter hypomethylation in CD8+ cells, reduced miR-21-5p with STAT3 upregulation, peri-lesional pre-lesional methylome shifts, and miR-9 methylation changes affecting adhesion cues (102–106)	Cytotoxic melanocyte killing, failure of regulatory balance, early niche instability, and altered adhesion/migration dynamics	Targeting CD8 cytotoxic programs and Treg/Teff balance, plus peri-lesional methylation biomarkers for progression or re-pigmentation
Alopecia Areata	PBMCs; scalp niche cells; CD14+ monocytes; NK cells; CD8+ T cells	Increased global DNA methylation and H3 acetylation, severity-linked chromatin changes, scalp enhancer-gene maps, peripheral scATAC remodeling in effector subsets, and elevated HDAC1 (80, 109–111)	Immune-privilege collapse, cytotoxic activation, interferon signaling, and loss of regulatory restraint	Immune stratification around interferon/JAK/STAT and cytotoxic programs, with HDAC-axis biomarker development

### 5.1 Psoriasis

Psoriasis is a common immune-mediated dermatosis affecting approximately 3.0% of adults in the United States, with incidence increasing with age and peaking in older populations (81, 82). Human lesional skin studies demonstrate extensive DNA methylation remodeling, including lesion-wide differentially methylated regions, enrichment of methylation signals within psoriasis susceptibility loci, and changes linked to the cardinal histopathologic features of plaque lesions (59, 83, 84). Complementing these methylome findings, lesional skin exhibits disease-specific enrichment of H3K27 acetylation at overexpressed genes, while studies of sorted immune-cell populations show that both CD4+ and CD8+ T cells harbor disease-associated methylation signatures that distinguish psoriasis from healthy controls and, more recently, from psoriatic arthritis; in CD4+ T cells, interferon-related loci are particularly prominent and methylation scores correlate with clinical activity (58, 85–87). Together, these findings support a model in which stable chromatin remodeling reinforces both epithelial and immune components of disease, with lesional keratinocytes maintaining a hyperproliferative, inflammatory transcriptional state and pathogenic T-cell compartments acquiring methylation programs consistent with durable effector-memory behavior rather than transient activation (83, 84). Translationally, the most developed implication lies in biomarker development rather than established epigenetic therapy, as methylation profiles in CD4+ and CD8+ T cells can distinguish clinically relevant psoriatic phenotypes, and in CD4+ T cells, disease-associated methylation patterns partially normalize following IL-17 or TNF blockade, suggesting a role for epigenomic readouts in patient stratification and treatment monitoring (86). Among autoimmune skin diseases, psoriasis currently has the strongest human epigenomic evidence, supported by studies of clinically resolved lesions, paired pre- and post-treatment biopsies, longitudinal treatment cohorts, single-cell analyses, and chromatin accessibility profiling, providing the most compelling evidence for persistent inflammatory and epigenetic memory.

### 5.2 Atopic dermatitis

Atopic dermatitis is among the most prevalent inflammatory skin diseases worldwide, affecting roughly 101 million adults and 103 million children globally, with US survey data suggesting approximately 16.5 million affected adults and a substantial moderate-to-severe subset experiencing significant quality-of-life impairment and high rates of comorbid atopic disease (88). Its early-onset, often chronic trajectory and strong genetic and environmental underpinnings make it a particularly important target for mechanistic epigenetic investigation. Integrated human methylome-transcriptome studies established that AD-associated DNA methylation changes are tissue-specific and strongest in the epidermis, a finding reinforced by subsequent lesional skin case-control methylation profiling that highlighted the epidermal compartment as the site of maximal epigenetic divergence from healthy controls (89, 90). At the epithelial-immune interface, lesional keratinocytes show Thymic Stromal Lymphopoietin (TSLP) promoter demethylation linked to TSLP overexpression, identifying an epigenetically controlled alarmin circuit as an upstream driver of type 2 inflammation, while monocytes show FCER1G promoter demethylation linked to FcϵRI overexpression, connecting myeloid epigenetic changes to the amplification of IgE-mediated responses. Circulating skin-homing CD4+CLA+ memory T cells carry the richest immune-cell epigenetic signal identified to date in AD, with differential methylation across 40 genes, reduced methylation upstream of IL13 that correlates directly with higher IL13 expression, and 16 differentially expressed miRNAs targeting broad inflammatory signaling pathways (91, 92). These findings are consistent with coordinated epigenetic regulation across epithelial, myeloid, and lymphoid compartments involved in type 2 inflammation. Together, they suggest that epithelial alarmin signaling, IgE/FcϵRI-associated myeloid responses, and skin-homing memory T-cell programs may converge on a shared inflammatory axis rather than representing entirely independent pathological processes. The CLA+ T-cell compartment is especially relevant for investigating skin-homing memory responses because these circulating cells share features of cutaneous immune imprinting while remaining distinct from bona fide TRM cells. Their epigenetic signatures provide insight into disease-associated immune programming but should not be interpreted as direct evidence of skin-resident immune memory (89, 91–93). In translational terms, TSLP- and FcϵRI-linked methylation changes define tractable upstream circuits whose epigenetic accessibility may predict responsiveness to biologics targeting these pathways, while CLA+ T-cell methylation and miRNA signatures offer plausible pharmacodynamic biomarkers for trials aimed at interrupting persistent skin-homing memory inflammation, particularly given that the IL13 methylation-expression correlation provides a quantitative link between epigenetic state and effector output that may prove especially valuable for distinguishing deep immunological response from surface-level symptom control (91–93). Current evidence is derived primarily from cross-sectional human lesional skin and peripheral immune-cell studies, whereas longitudinal analyses of resolved lesions and mechanistic chromatin-level investigations remain comparatively limited.

### 5.3 Cutaneous lupus erythematosus

Cutaneous lupus erythematosus is an uncommon but clinically important disorder, with an overall incidence of 3.9 per 100,000 and an age- and sex-adjusted prevalence of 108.9 per 100,000 in long-term population-based data (94). CLE encompasses a heterogeneous spectrum of subtypes, including discoid, subacute, and acute forms, which differ in their relationship to systemic lupus erythematosus and in their risk of scarring and permanent tissue damage, making the interface between cutaneous and systemic disease a particularly complex diagnostic and therapeutic challenge. In discoid CLE, lesional microRNA profiling has identified miR-31 and miR-485-3p as key disease-associated regulators, while more recent human studies demonstrate that loss of miR-885-5p promotes NF-κB pathway activation and immune recruitment, directly linking noncoding RNA dysregulation to inflammatory amplification in lesional skin (95, 96). At the immune-cell level, naïve CD4+ T cells from patients with systemic lupus who exhibit malar or discoid rash display cutaneous-phenotype–specific methylation signatures enriched in antigen-processing, TAP-dependent cross-presentation, and MHC class I pathways, suggesting that epigenetic reprogramming of adaptive immune cells aligns with the interface-directed inflammation characteristic of cutaneous disease (97, 98). Complementing these immune findings, keratinocytes in lupus show Hippo-pathway methylation abnormalities centered on WWC1 that increase UVB-sensitive apoptosis, providing a molecular epigenetic basis for the photosensitivity that defines CLE (97, 98). Together, these human data support a two-compartment model in which lesional epithelial cells are epigenetically primed for photosensitive injury and inflammatory mediator release, while adaptive immune cells are simultaneously skewed toward enhanced antigen handling and interface-directed effector activity. These processes may be mutually reinforcing, as keratinocyte apoptosis driven by epigenetically sensitized Hippo signaling generates antigenic substrates that reprogrammed T cells are poised to amplify (95–98). Because direct, cell-resolved epigenomic profiling of skin-resident immune populations in CLE remains limited, current mechanistic inference relies on integrating lesional tissue and circulating-cell datasets, highlighting an important gap for future investigation (95–98). Translationally, the miR-31/miR-485-3p, miR-885-5p/NF-κB, antigen-presentation, and photosensitivity/Hippo axes identify human-defined pathways that may inform future therapeutic strategies, with WWC1-centered Hippo methylation abnormalities offering a particularly compelling molecular explanation for UVB-triggered disease activity and a potential target for mitigating photosensitive flares (95–98). Current mechanistic understanding relies largely on cross-sectional human lesional tissue and peripheral immune-cell studies, with relatively limited cell-resolved epigenomic analyses of skin-resident immune populations.

### 5.4 Vitiligo

Vitiligo affects an estimated 0.76% to 1.11% of US adults, with systematic review data indicating incidence estimates ranging from 24.7 to 61.0 cases per 100,000 person-years across population-based studies (99, 100). Vitiligo is characterized by targeted melanocyte destruction by immune effectors, positions it as a tractable model for studying chromatin-regulated cytotoxic immunity in the skin (101). Early human studies identified abnormal global DNA methylation in peripheral blood mononuclear cells, establishing a systemic epigenetic footprint extending beyond lesional skin (102, 103). Subsequent mechanistic work demonstrated that CD8+ T cells from patients with vitiligo exhibit perforin promoter hypomethylation linked to PRF1 overexpression, with corresponding evidence that these epigenetically altered cytotoxic cells directly mediate melanocyte injury, thereby connecting chromatin state to the principal effector mechanism of pigment loss (102, 103). A complementary immune axis involves dysregulated immune balance: miR-21-5p is reduced and STAT3 is increased in patient CD4+ T cells, and restoration of miR-21-5p shifts Th17/Th22-polarized cells toward a more regulatory phenotype while indirectly protecting melanocytes from apoptosis, identifying a noncoding RNA–dependent checkpoint in Teff/Treg regulation relevant to disease control (104). At the tissue level, a 2024 skin methylome study identified 264 differentially methylated and expressed genes in lesional and peri-lesional skin, including 16 “pre-DMEGs” in peri-lesional tissue that appear epigenetically altered before full transcriptional conversion, while a separate study demonstrated that miR-9 methylation in keratinocytes regulates E-cadherin and β1-integrin signaling relevant to melanocyte adhesion and UVB-induced re-pigmentation (105, 106). Collectively, these findings suggest that vitiligo represents an epigenetically primed cytotoxic niche in which both immune effectors and the peri-lesional epidermal microenvironment are reprogrammed prior to overt depigmentation. The convergence of PRF1-high CD8+ T-cell activity with miR-21–STAT3–mediated failure of regulatory restraint indicates that two interacting epigenetic programs, enhanced cytotoxic capacity and impaired immune regulation, may need to be addressed simultaneously for durable disease control (103–105). The identification of pre-DMEGs in peri-lesional skin further suggests that the window for epigenetic intervention may extend into clinically uninvolved tissue before irreversible melanocyte loss occurs, with direct implications for trial design. Translationally, these data highlight three key entry points: attenuation of cytotoxic CD8+ programs centered on PRF1, restoration of miR-21–STAT3–regulated Treg/Teff balance, and use of peri-lesional methylation signatures as biomarkers of epigenetically unstable skin that may be prone to progression or, under treatment, to re-pigmentation (103–106). Overall, the evidence base for vitiligo is derived primarily from cross-sectional human skin methylome studies and peripheral immune-cell analyses, providing growing support for disease-associated epigenetic dysregulation. However, most available studies remain cross-sectional, and direct longitudinal evidence linking persistent epigenetic programs to tissue-resident immune memory and long-term clinical recurrence remains limited.

### 5.5 Alopecia areata

Alopecia areata is a common autoimmune hair disorder characterized by non-scarring, often patchy hair loss driven by collapse of immune privilege at the hair follicle, with a lifetime incidence of approximately 2.11% and US diagnosed incidence rates near 87 to 93 per 100,000 person-years in recent analyses, with evidence suggesting disease burden may be increasing over time (107, 108). AA spans a broad clinical spectrum from limited patchy disease to total scalp or body hair loss, and its variable course makes biological stratification a pressing clinical need that epigenetic profiling is now beginning to address. Early human PBMC studies demonstrated increased global DNA methylation, increased histone H3 acetylation, reduced H3K4 methylation, and widespread dysregulation of chromatin-modifying enzymes, with H3 acetylation correlating with both disease severity and CCL5/RANTES expression, establishing a link between epigenetic remodeling and inflammatory activity (109). More recent cell-resolved approaches have advanced this framework: a 2023 single-cell scalp multiomic atlas generated matched chromatin accessibility and transcriptomic profiles from healthy and AA scalp, mapping hair-follicle niche cell states and providing a tissue-anchored reference for disease-associated epigenetic alterations (80). Building on this, a 2025 peripheral immune multiome study identified 42,248 significant ATAC peaks, demonstrating pronounced epigenetic remodeling in CD14+ monocytes, NK cells, and CD8+ T cells, with severity-dependent patterns most evident in patients with extensive disease; open chromatin in severe AA was enriched at loci governing antigen presentation, chemokine signaling, and interferon responses, alongside high expression of cytotoxic mediators including PRF1 and GNLY (110). Complementary work has also identified elevated serum HDAC1 levels in AA, extending the histone-regulatory signal to a potentially measurable biomarker (111). Collectively, these data support a model in which AA progression reflects selective epigenetic activation of cytotoxic-memory and innate effector programs, coupled with relative failure of regulatory circuits, linking local immune-privilege collapse at the hair follicle to systemic immune priming detectable in peripheral blood. The severity dependence of chromatin accessibility patterns suggests that epigenetic remodeling is progressive rather than binary, deepening with increasing clinical extent and potentially enabling epigenomic staging of disease trajectory (80, 109, 110). Among inflammatory dermatoses, AA provides particularly strong human evidence that chromatin state and effector-cell identity are tightly coupled across both tissue and peripheral compartments. Translationally, the key pathways identified, including interferon–JAK/STAT signaling, antigen presentation, NK-cell activity, and cytotoxic CD8 programs, align closely with the mechanism of action of JAK inhibitors, an approved therapeutic class for AA, providing a direct link between epigenomic findings and treatment rationale. Elevated HDAC1 further suggests that chromatin-modifying enzymes may serve as pharmacodynamic biomarkers, supporting blood-based monitoring of treatment response and positioning AA as a model in which epigenetic profiling can inform stage-specific immune stratification and guide therapeutic decision-making (109–111). Recent single-cell multiomic studies have substantially strengthened the human evidence base for alopecia areata. However, compared with psoriasis, direct evidence linking persistent epigenetic programs to tissue-resident immune memory and long-term clinical recurrence remains limited.

## 6 Environmental and microbial drivers of epigenetic change

### 6.1 Ultraviolet radiation

Ultraviolet (UV) radiation represents a prototypical environmental exposure through which acute tissue injury is translated into durable changes in gene regulation. In keratinocytes, solar-simulated UV exposure induces rapid alterations in chromatin state, most prominently through modulation of histone post-translational modifications that regulate transcriptional accessibility. Studies of chronically sun-exposed human skin demonstrate increased histone H3 acetylation alongside altered expression of histone acetyltransferases and deacetylases, indicating that repeated UV exposure not only modifies chromatin structure but also reprograms the enzymatic machinery that maintains it (112–114). These changes are associated with sustained activation of stress-response and inflammatory pathways, supporting a role for histone modifications in mediating persistent UV-induced transcriptional programs (115–117).

In contrast, acute UVB exposure does not consistently produce widespread genome-wide shifts in DNA methylation. Instead, methylation changes tend to be selective and temporally delayed, affecting loci involved in immune regulation, apoptosis, and epidermal homeostasis (114, 115). This distinction supports a model in which histone modifications enable rapid and reversible transcriptional responses to UV injury, whereas DNA methylation contributes to longer-term stabilization of these responses under conditions of repeated exposure. Accordingly, chronic UV exposure is more likely to establish persistent epigenetic states than isolated acute injury.

This temporal distinction has direct relevance for autoimmune disease. Acute UV exposure induces transient activation of type I interferon pathways, whereas repeated exposure promotes sustained chromatin remodeling, cumulative tissue damage, and prolonged immune activation. These processes may lower the threshold for subsequent inflammatory responses and thereby contribute to disease persistence in susceptible individuals.

LCs provide a clear example of environmentally driven immune reprogramming. UV radiation reduces epidermal LC density and impairs antigen-presenting capacity, contributing to local immunosuppression (118, 119). However, surviving or migrating LCs can adopt altered functional states, including the promotion of tolerogenic immune responses in draining lymph nodes (120, 121). More recent work suggests that LC function exists along a spectrum of tolerogenic and immunogenic states shaped by local epidermal signals and underlying gene-regulatory networks (122). Although direct epigenomic mapping of UV-exposed human LCs remains limited, these functional changes are consistent with the possibility of durable alterations in chromatin accessibility and transcriptional control.

UV exposure is particularly relevant in CLE, where it serves as a well-established disease trigger. UVB-induced DNA damage activates cytosolic nucleic acid sensing pathways, including cGAS signaling, leading to robust production of type I interferons (116). In parallel, UV-damaged keratinocytes promote recruitment of plasmacytoid dendritic cells (pDCs), in part through chemerin-mediated signaling (123, 124). As major producers of type I interferons, pDCs amplify local inflammation, and their depletion has been shown to reduce interferon signaling and improve disease severity in CLE (125).

Clinically, these findings explain why UV radiation functions as both a trigger and a disease-maintaining factor in photosensitive autoimmune conditions. Acute exposure induces transient immune activation, whereas repeated exposure reinforces chronic inflammatory circuits and epigenetic remodeling. Consistent with this model, strict photoprotection reduces interferon-driven inflammation in CLE, highlighting UV radiation as a modifiable driver of disease activity (126, 127).

### 6.2 Skin microbiome

The skin is colonized by diverse microbial communities that vary by anatomical site rather than forming a uniform distribution. Sebaceous, moist, and dry regions harbor distinct microbial populations, with dominant genera including *Cutibacterium, Corynebacterium*, and *Staphylococcus*. This topographic organization is highly reproducible across individuals and reflects a stable host–microbe equilibrium (128–130). Beyond passive colonization, commensal microbes actively shape cutaneous immunity, promote epidermal differentiation, and support barrier repair, establishing the microbiome as a functional component of the skin microenvironment.

A key mechanism linking microbes to host gene regulation is the production of metabolites with epigenetic activity. Short-chain fatty acids (SCFAs), generated through microbial metabolism, can modulate chromatin state by inhibiting histone deacetylases (HDACs), thereby altering transcriptional programs. In skin-specific models, *Cutibacterium acnes*–derived SCFAs inhibit HDAC8 and HDAC9 in keratinocytes, leading to measurable changes in inflammatory gene expression (131, 132). These findings provide mechanistic evidence that microbial metabolites can influence host epigenetic regulation and link microbiome composition to chromatin-level control of inflammation.

Dysbiosis is most clearly characterized in AD where disease flares are associated with reduced microbial diversity and dominance of *Staphylococcus aureus*. Longitudinal studies suggest that microbial shifts can precede clinical disease onset, indicating that the microbiome may influence disease susceptibility rather than simply reflecting barrier disruption (133–135). Mechanistically, dysbiosis may amplify inflammatory signaling while altering exposure to microbiome-derived epigenetic modulators. However, direct links between AD-associated microbial changes and specific DNA methylation or histone modification patterns in human skin remain limited.

In psoriasis, microbiome alterations are less consistent but remain biologically relevant. While specific taxa vary across studies, emerging evidence suggests that host–microbe interactions contribute to disease severity, with certain organisms, such as *Corynebacterium simulans*, associated with inflammatory pathway activation (136–138). These findings raise the possibility that microbial composition may prime the skin for inflammation and contribute to disease heterogeneity.

These insights have direct therapeutic implications. Symbiotic microbial strains derived from healthy skin can suppress *S. aureus* colonization and restore aspects of microbial balance in AD. Early clinical studies demonstrate that topical *Roseomonas* mucosa reduces disease severity and steroid use, while *Staphylococcus hominis* A9 has emerged as a targeted bacteriotherapy (139–141). Whether such interventions can durably reprogram pathogenic immune states or reverse established epigenetic memory remains an open question.

### 6.3 Mechanical injury and the Koebner phenomenon

The Koebner phenomenon refers to the development of new lesions of an existing dermatosis in previously unaffected skin following trauma. In psoriasis, this can occur after diverse forms of injury, including cuts, surgical wounds, burns, tattoos, and chronic friction or pressure. Clinical studies indicate that a substantial subset of patients exhibit this trauma-responsive pattern, demonstrating that lesion formation can be triggered by local injury independent of systemic disease activity (142, 143).

An epigenetic basis for this phenomenon is increasingly supported. Experimental studies show that prior inflammation can establish a persistent chromatin-based memory in epithelial stem cells, enabling more rapid and robust responses to subsequent injury (144). Consistent with this, clinically resolved psoriatic skin retains residual transcriptomic and epigenetic alterations, suggesting that disease-associated regulatory states persist even after visible inflammation subsides (17, 145). These findings support a model in which previously affected skin remains epigenetically primed, lowering the threshold for disease reactivation.

Mechanical stress itself is biologically active. Stretch and injury activate mechano-transduction pathways in keratinocytes, including YAP/TAZ and AP-1 signaling, which regulate genes involved in proliferation, wound repair, and inflammation. These pathways interact with chromatin remodeling machinery to maintain accessibility at key regulatory loci, promoting a pro-inflammatory transcriptional state (146, 147). Despite these advances, a trauma-specific epigenomic map of human Koebner lesions remains lacking.

Clinically, this framework may help explain why psoriasis lesions frequently arise in surgical scars, tattoo sites, and areas of repeated mechanical stress. Rather than representing entirely new disease events, these lesions likely reflect reactivation of pre-existing pathogenic programs in epigenetically primed skin (148–150).

### 6.4 Metabolic inputs

Cellular metabolism is a direct regulator of epigenetic state, as metabolites serve as substrates, cofactors, and inhibitors of chromatin-modifying enzymes. Acetyl-CoA provides the acetyl groups required for histone acetylation, while α-ketoglutarate supports the activity of DNA and histone demethylases. In contrast, metabolites such as succinate and fumarate inhibit these enzymes, promoting accumulation of methylation marks (151–154). Through these mechanisms, metabolic pathways are tightly coupled to transcriptional regulation and immune cell function.

In psoriasis, this coupling is particularly evident. Increased glutaminolysis in psoriatic T cells elevates acetyl-CoA levels and enhances histone H3 acetylation at the IL17A locus, directly linking metabolic state to effector cytokine production (155). Additional studies demonstrate increased citrate-derived acetyl-CoA and lipogenic programming in IL-17–producing γδ T cells, reinforcing pro-inflammatory transcriptional programs (156).

Hypoxia represents another key metabolic signal. Many histone demethylases, including Jumonji-domain enzymes, require oxygen as a cofactor; reduced oxygen availability limits their activity and alters histone methylation patterns (157, 158). In psoriatic skin, increased expression of hypoxia-inducible factor 1α (HIF-1α) suggests that local hypoxia contributes to stabilization of inflammatory gene expression (159, 160).

Obesity further illustrates the systemic dimension of metabolic–epigenetic regulation. Higher body mass index is consistently associated with increased risk and severity of psoriasis (161, 162). Although direct epigenetic mapping in human skin remains limited, obesity-associated metabolic and inflammatory changes likely influence immune cell chromatin states, contributing to disease persistence (163).

### 6.5 Oxidative stress

Reactive oxygen species (ROS) represent a key interface between environmental stress and epigenetic regulation. ROS influence gene expression through DNA damage, activation of redox-sensitive signaling pathways, and modulation of epigenetic enzymes. In particular, oxidative stress can alter DNA methylation by affecting DNA methyltransferase activity, demethylation processes, and availability of methyl donors, leading to both locus-specific and global changes in methylation patterns (164, 165).

Vitiligo provides a clear example of oxidative stress–driven immune dysregulation. ROS accumulation is thought to precede and promote immune-mediated melanocyte destruction. Keratinocytes exposed to oxidative stress recruit pathogenic CD8^+^ T cells through the CXCL16–CXCR6 axis, linking local redox imbalance to adaptive immune activation (166–168).

Recent work has identified a direct epigenetic mechanism underlying this process. ROS exposure induces hypermethylation of the USP18 promoter in keratinocytes and melanocytes, leading to increased ISG15 expression and enhanced IFN-γ production by CD8^+^ T cells (169). This provides a mechanistic link between oxidative stress, DNA methylation, and sustained inflammatory signaling.

Additional studies show that ROS-induced release of HMGB1 from melanocytes promotes immune activation and inflammation, further amplifying disease progression (170). Together, these findings support a model in which oxidative stress acts as both an initiating and amplifying factor, driving epigenetic reprogramming and persistent immune activation in vitiligo.

## 7 Emerging technologies in epigenetic profiling

Emerging epigenomic technologies are rapidly transforming the study of inflammatory skin disease by enabling analysis of gene regulation at cellular, spatial, and functional resolution. While each modality provides distinct insights, their combined use is increasingly enabling more mechanistic investigation of disease-relevant regulatory circuits. In dermatology, these approaches are particularly powerful because molecular findings can be directly linked to tissue architecture, histopathology, and longitudinal clinical phenotypes. At the same time, the unique biological and technical characteristics of skin present important considerations that influence study design and data interpretation. Key platforms, their capabilities, and limitations are summarized in **Table 5**.

**Table 5:** Emerging epigenomic technologies in skin biology.

Technology	What it measures	Strength	Limitation	Skin-specific insight	References
scATAC-seq	Chromatin accessibility	Cell-type resolution of regulatory elements	Low cell yield; sparse signal	Identifies regulatory programs in TRM and lesional immune cells	(11, 172, 174–176)
Multiome (RNA + ATAC)	Gene expression + chromatin accessibility	Direct enhancer–gene linkage within the same cell	High cost; computational complexity	Maps disease-specific regulatory circuits	(80, 172, 180–182)
Single-cell methylation (scNOMe-seq, scNMT-seq)	DNA methylation ± accessibility ± RNA	Multi-layer epigenetic integration	Low throughput; technically demanding	Defines stable epigenetic states	(174, 175)
Spatial transcriptomics (Visium, MERFISH, Xenium, CosMx)	Gene expression in tissue context	Preserves spatial architecture	Resolution limits (platform-dependent)	Identifies inflammatory micro-niches	(27, 177–179)
Spatial epigenomics (spatial ATAC, CUT&Tag)	Chromatin state in situ	Links regulation to tissue location	Early-stage; limited datasets	Emerging in skin disease	(172–174)
CUT&Tag/CUT&RUN	Histone marks/TF binding	Low input; compatible with biopsies	Limited genome-wide breadth	Enables chromatin profiling from small samples	(173)
Multi-omics integration (ArchR, Seurat, Tangram)	Integrated regulatory networks	Mechanistic inference across modalities	Alignment challenges; data sparsity	Reconstructs tissue-level regulatory circuits	(180–182)
Epigenome editing (dCas9-based tools)	Functional perturbation of chromatin	Tests causality	Delivery and safety challenges	Future therapeutic applications	(183, 184)

### 7.1 Considerations for epigenomic studies in skin

The skin presents both unique advantages and methodological challenges for epigenomic investigation. Unlike many internal organs, cutaneous tissue is routinely sampled in clinical practice, generating extensive collections of archived specimens linked to detailed histopathologic interpretation and longitudinal clinical outcomes (171). These resources provide a valuable foundation for retrospective studies aimed at connecting molecular signatures with disease phenotype, progression, and treatment response. However, several features of skin complicate epigenomic analysis. Tissue processing methods commonly used in dermatopathology may restrict the application of certain assays that require intact chromatin architecture (172, 173), although recent FFPE-compatible approaches have begun to address some of these limitations (174). In addition, the structural complexity of skin, including tightly adherent cellular compartments and extracellular matrix components, can make isolation of viable single cells technically challenging. Nucleic acid quality may also be affected by tissue-specific factors that complicate transcriptomic analyses. At the same time, the highly organized arrangement of epidermal, dermal, adnexal, vascular, and immune-cell niches means that preservation of spatial context is often critical for biological interpretation. These considerations have shaped the development and application of emerging technologies in cutaneous research and help explain the growing interest in spatially resolved and low-input epigenomic approaches.

### 7.2 Single-cell epigenomics

Single-cell epigenomic technologies resolve a major limitation of bulk profiling by capturing chromatin state at cellular resolution within heterogeneous skin lesions. Approaches such as scATAC-seq and RNA–ATAC multi-omics link regulatory element accessibility to gene expression within individual cells, enabling inference of cell-specific regulatory circuits (172, 174, 175). In human skin, these methods are beginning to define disease-relevant cell states and enhancer–gene relationships, although their application remains limited by low biopsy yield and technical complexity (11, 172, 176). Recent advances in single-cell FFPE-compatible ATAC-seq have enabled chromatin accessibility profiling of archived clinical specimens, expanding opportunities for retrospective epigenomic studies (177).

### 7.3 Spatial transcriptomics and spatial epigenomics

Spatial technologies address a critical limitation of dissociation-based assays by preserving tissue architecture. In skin, where function depends on precise spatial organization across epidermal and dermal compartments, these approaches reveal localized cellular interactions that are not captured by bulk or single-cell analyses alone. Spatial transcriptomics has identified discrete inflammatory micro-niches and coordinated immune–stromal signaling networks in diseases such as psoriasis and AD (178–180). More recently, spatial epigenomic technologies, including spatial ATAC-seq and spatial CUT&Tag, have enabled mapping of chromatin accessibility and histone-modification landscapes within tissue sections (181, 182). However, most current studies remain limited to fresh or frozen tissues and rely on integration with single-cell transcriptomic datasets for cell-type resolution and regulatory inference.

### 7.4 Multi-omics integration

The primary value of emerging epigenomic technologies lies in their integration. Computational frameworks enable joint analysis of transcriptomic and chromatin accessibility data, allowing inference of gene-regulatory relationships and projection of these states into tissue space (183, 184). In inflammatory skin disease, integrated analyses have revealed spatially organized inflammatory programs and defined cell–cell interaction networks that would not be resolved by single modalities alone (179, 185). Despite these advances, challenges remain, including data sparsity, cross-sample alignment, and limited longitudinal datasets.

### 7.5 Epigenome editing

Epigenome editing extends epigenetic profiling into functional manipulation, enabling direct testing of causal regulatory mechanisms. dCas9-based systems can be fused to epigenetic effectors to activate or repress gene expression at specific loci, allowing interrogation of disease-associated regulatory elements (186). While these approaches have demonstrated strong potential in experimental systems, clinical application in dermatology remains limited by challenges in delivery, specificity, and long-term safety (187). Nevertheless, given the accessibility of skin, epigenome editing represents a promising future strategy for targeted therapeutic modulation of pathogenic gene regulation.

## 8 Therapeutic targeting of epigenetic pathways

Epigenetic therapies may provide a mechanistically distinct approach to inflammatory skin disease by targeting the regulatory systems that sustain pathogenic gene expression rather than individual downstream cytokines. While most clinical experience remains rooted in oncology, emerging data suggest that modulation of chromatin state can influence both immune and epithelial compartments, offering a potential strategy to alter disease persistence at its source. A summary of major epigenetic therapeutic classes, their mechanisms, clinical status, and key limitations in dermatology is provided in **Table 6**.

**Table 6:** Epigenetic therapeutic agents in dermatology.

Drug class	Agents	Dermatology application	Limitations	References
HDAC inhibitors	Vorinostat; romidepsin	Approved for cutaneous T-cell lymphoma; preclinical in psoriasis and other inflammatory dermatoses	Systemic toxicity; broad transcriptional effects; limited autoimmune data	(68, 185–190)
DNMT inhibitors	5-azacytidine; decitabine	Preclinical evidence in psoriasis and inflammatory models	Limited clinical data; lack of cell-type specificity; systemic toxicity concerns	(179)
BET inhibitors	JQ1; OTX015 (birabresib)	Preclinical activity in psoriasiform inflammation	Limited dermatology translation; tolerability and specificity concerns	(181)
EZH2 inhibitors	Tazemetostat; GSK126	Preclinical in psoriasis; clinically validated in oncology	Limited dermatology data; systemic effects may exceed therapeutic need	(192)
Epigenome editing	dCas9-DNMT3A; dCas9-p300; dCas9-TET1	Preclinical; potential for locus-specific reprogramming	Delivery challenges; off-target effects; early translational stage	(183, 184)

### 8.1 Histone deacetylase inhibitors

Histone deacetylase inhibitors represent the most clinically advanced epigenetic therapies in dermatology and provide the clearest proof of principle that chromatin-directed treatment can be effective in human skin disease. Vorinostat and romidepsin are approved for cutaneous T-cell lymphoma (CTCL), where they demonstrated meaningful activity in heavily pretreated populations (188–190). Although these data derive from lymphoma rather than autoimmune disease, they establish that modulation of histone acetylation can produce clinically meaningful cutaneous effects. Mechanistically, HDAC inhibition affects both immune and epithelial compartments. In psoriasis models, topical inhibitors reduce interleukin-17–driven inflammation, attenuate dendritic cell activation, and promote keratinocyte differentiation (191, 192). These dual effects highlight the potential for chromatin-directed therapy to simultaneously target inflammatory circuits and barrier dysfunction. Despite strong mechanistic support, translation beyond CTCL remains limited. Systemic toxicities and lack of specificity remain key barriers, supporting a shift toward topical or skin-restricted delivery strategies. The accessibility of cutaneous lesions may facilitate localized delivery approaches, allowing modulation of disease-relevant pathways within affected tissue while potentially reducing systemic exposure (68, 193).

### 8.2 DNA methyltransferase inhibitors

DNA methyltransferase inhibitors directly target the maintenance of pathological methylation states and represent a strategy for modifying stable epigenetic programming (**Table 6**). Preclinical studies in psoriasis demonstrate that DNMT inhibition can suppress keratinocyte proliferation and modulate inflammatory signaling, supporting a role in both epidermal and immune regulation (179). Broader immunologic data further suggest that DNMT inhibition can attenuate autoimmune responses and promote regulatory T-cell populations. However, translation into dermatology remains early, with limited clinical data and ongoing concerns regarding systemic exposure and lack of cell-type specificity.

### 8.3 Bromodomain and extra-terminal domain inhibitors

BET inhibitors target epigenetic “reader” proteins that interpret acetylated chromatin and sustain transcription of inflammatory genes. Preclinical studies in psoriasis demonstrate that BET inhibition suppresses Th17-associated pathways and reduces disease severity in experimental models (194). Despite strong mechanistic rationale, clinical development in dermatology remains limited. Current efforts are focused on improving specificity and tolerability, including development of bromodomain-selective inhibitors and exploration of localized delivery strategies.

### 8.4 Enhancer of zeste homolog 2 inhibitors

EZH2 inhibitors block deposition of the repressive histone mark H3K27 trimethylation and represent a complementary approach to chromatin modulation. In psoriasis, EZH2 activity is increased in lesional skin, and inhibition reduces keratinocyte proliferation and improves disease severity in preclinical models (195). Although clinical validation exists in oncology, translation to autoimmune dermatology remains limited. Future applications will likely depend on targeted delivery and improved patient selection.

### 8.5 Reprogramming immune memory: a forward-looking perspective

Although current clinical evidence for epigenetic therapies in autoimmune skin disease remains limited, preclinical studies and experience in cutaneous T-cell lymphoma provide proof of principle that modulation of chromatin state is feasible, supporting continued investigation rather than established clinical application. Collectively, the therapeutic classes summarized in **Table 6** support a broader conceptual shift: chronic inflammatory skin disease may persist not only through ongoing cytokine signaling, but also through persistent epigenetic programs within tissue-resident immune cells that may contribute to disease chronicity. This framework suggests that effective therapy may require both suppression of active inflammation and disruption of underlying chromatin-based memory. Accordingly, epigenetic therapies, particularly in combination with cytokine-targeted agents, represent a promising investigational strategy that may improve the durability of response and potentially reduce recurrence but require validation in future clinical studies. An important consideration for future therapeutic development is the balance between efficacy and biological specificity. Because the skin is readily accessible, dermatology offers a unique setting in which chromatin-modifying agents could potentially be delivered directly to affected tissue, reducing the need for systemic exposure. At the same time, epigenetic pathways govern fundamental processes extending beyond inflammation, including cell fate decisions, tissue renewal, and maintenance of epidermal homeostasis. As a result, therapeutic modulation of these pathways will likely require careful control over target selection, timing, and duration of treatment. Advances in cell-selective targeting, localized delivery platforms, and transient epigenetic editing approaches may help maximize therapeutic benefit while minimizing unintended effects on normal tissue function.

## 9 Discussion

### 9.1 Autoimmune skin disease as a disorder of maladaptive epigenetic memory

A unifying insight emerging from this review is that maladaptive epigenetic memory may provide an important conceptual framework for understanding the chronicity, site-specificity, and recurrence of autoimmune skin disease. Rather than replacing the established roles of genetic susceptibility and ongoing antigen exposure, this framework complements them by helping explain how prior inflammatory events may contribute to persistent, site-specific disease recurrence. While genetic risk loci define a permissive landscape and cytokine signaling drives acute inflammation, these factors do not fully account for the persistence of disease in clinically resolved skin, the localization of lesions to previously affected sites, or the variability in treatment response. Instead, converging evidence across multiple diseases, including psoriasis, AD, vitiligo, and CLE, supports a model in which prior inflammatory events may become encoded into persistent chromatin states within both epithelial and immune compartments. The strongest support for this framework currently comes from psoriasis and vitiligo, where tissue-resident memory T cells and persistent disease-associated transcriptional programs have been associated with lesion recurrence and site-specific relapse (196–198). Similar mechanisms have been proposed in AD, CLE, and AA based on emerging epigenetic and immunologic observations, although direct evidence implicating resident-memory programs in long-term disease persistence remains less well established (199–201). Tissue-resident memory T cells, keratinocytes, and stromal cells retain disease-associated transcriptional and epigenetic programs even after apparent clinical resolution, consistent with a form of biological memory that may predispose the tissue to rapid reactivation upon subsequent stimulation. Collectively, these observations support the concept that autoimmune skin disease may be viewed not only as a disorder of immune activation but also as one involving dysregulated cellular memory embedded within chromatin architecture.

### 9.2 A multi-hit model of epigenetic reprogramming in skin autoimmunity

**Figure 2:**
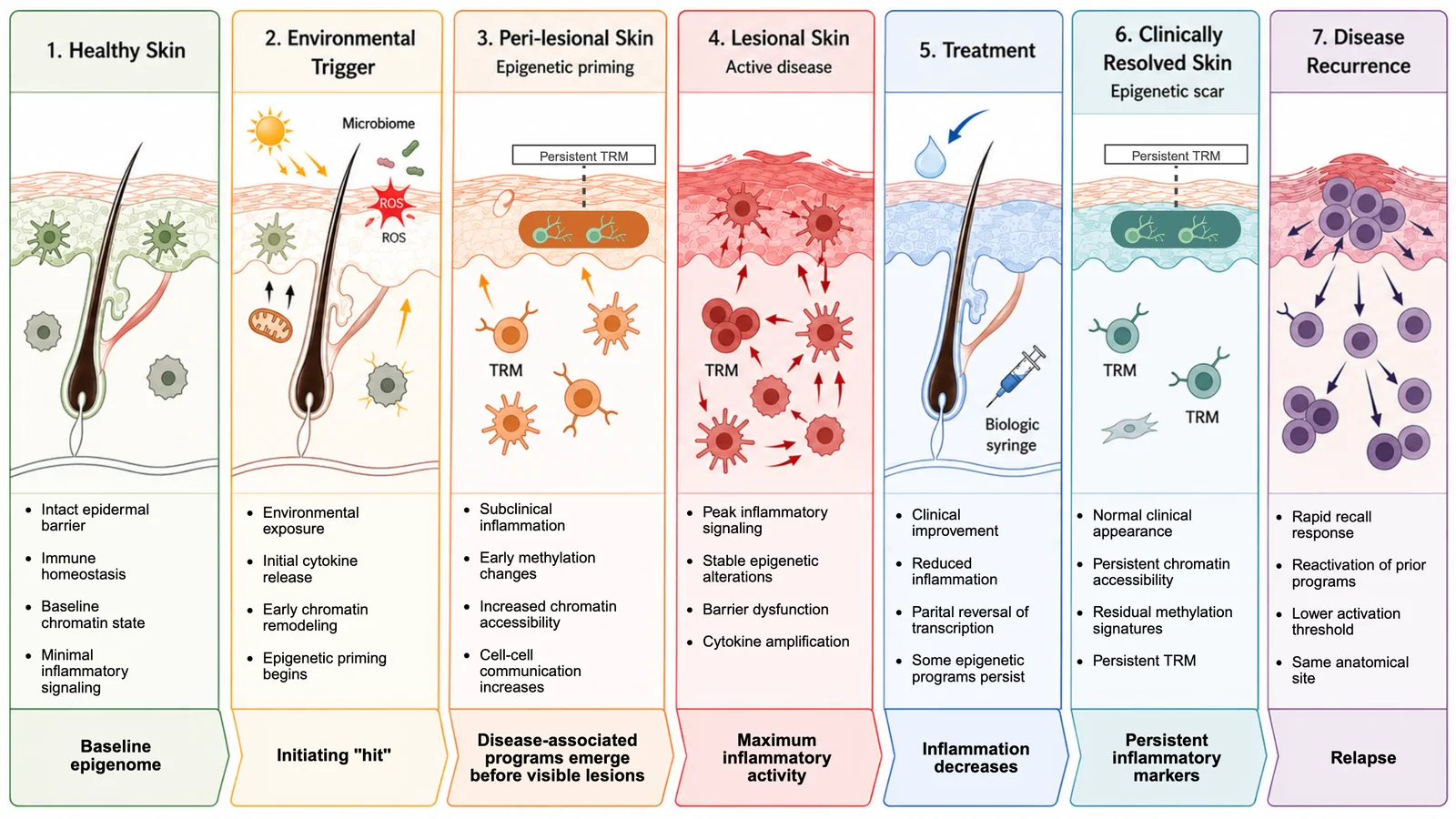
Longitudinal evolution of epigenetic memory in autoimmune skin disease. Proposed model illustrating the progression from healthy skin through environmental triggering, peri-lesional epigenetic priming, active lesional disease, treatment, clinically resolved skin, and disease recurrence. Repeated environmental and inflammatory exposures are hypothesized to induce progressive epigenetic reprogramming, including alterations in DNA methylation, histone modifications, chromatin accessibility, and cell-specific transcriptional programs. Although clinical inflammation may resolve following treatment, persistent epigenetic alterations within tissue-resident memory T (TRM) cells, keratinocytes, and other skin-resident cell populations may constitute an epigenetic “scar” that lowers the threshold for subsequent disease reactivation at the same anatomical site. The lower panel summarizes the proposed temporal evolution of major molecular and cellular features across disease stages and highlights opportunities for future longitudinal, multi-omic studies to evaluate this model.

The data reviewed here support a multi-hit model in which disease emerges from the interaction of genetic predisposition, cell-type–specific regulatory architecture, and environmental exposures that collectively shape epigenetic state. In this framework, genetic risk variants establish a permissive chromatin landscape that influences enhancer selection and transcription factor binding. Environmental inputs, including ultraviolet radiation, microbial signals, mechanical injury, metabolic shifts, and oxidative stress, may act as epigenetic “hits” that remodel chromatin accessibility, histone modifications, and DNA methylation patterns. Over time, repeated exposures consolidate these changes into stable regulatory programs, resulting in persistent transcriptional reprogramming of both immune and epithelial cells. This model provides a more comprehensive explanation of clinical epidemiology than purely genetic or cytokine-centered paradigms. It accounts for incomplete penetrance of genetic risk, the importance of environmental triggers, and the relapsing–remitting nature of disease. Importantly, this model may help explain why lesions recur at specific anatomical sites, as tissue-resident cells within these regions retain epigenetically encoded inflammatory programs that can be reactivated. A conceptual overview of this proposed multi-hit model and the progression from environmental exposure to persistent inflammatory memory and disease recurrence is illustrated in **Figure 2**.

Future longitudinal multi-omic studies could test this model by serially sampling lesional, peri-lesional, clinically resolved, and distant non-lesional skin before treatment, after clinical resolution, and during disease recurrence. Integrating single-cell chromatin accessibility, DNA methylation, transcriptomic, and spatial datasets would help determine whether disease-associated regulatory programs arise before visible lesion formation, persist after inflammation resolves, and become reactivated during relapse. Paired longitudinal sampling from the same anatomical site would be particularly informative for assessing whether cumulative environmental or inflammatory exposures progressively reinforce stable, cell-specific chromatin states and lower the threshold for subsequent disease reactivation.

### 9.3 Defining the missing links in cutaneous epigenetic regulation

Despite significant advances, several critical gaps remain in our understanding of epigenetic regulation in autoimmune skin disease. First, there is a lack of direct, cell-type–resolved epigenomic data from human skin, particularly for tissue-resident immune populations such as TRM cells, dendritic cells, and macrophages. Much of the current mechanistic understanding is inferred from bulk tissue analyses or circulating immune cells, which may not fully capture the regulatory complexity of the skin microenvironment. Second, the temporal dynamics of epigenetic change remain poorly defined. It is unclear which alterations represent early, reversible adaptations versus stable, disease-sustaining programs. The identification of peri-lesional epigenetic changes in conditions such as vitiligo suggests that pathogenic reprogramming may precede overt clinical disease, but this has not been systematically studied across disorders. Third, the relationship between different layers of epigenetic regulation, including DNA methylation, histone modification, chromatin accessibility, and three-dimensional genome organization, remains incompletely integrated in human disease. While emerging multi-omic approaches are beginning to address this gap, most studies remain cross-sectional and limited in scale, underscoring the need for longitudinal, paired-sampling designs to establish persistent cell-intrinsic epigenetic memory. While associations between chromatin state and gene expression are well documented, direct causal evidence linking specific epigenetic alterations to disease persistence in humans remains limited. Current support for causal mechanisms is derived primarily from complementary functional studies in experimental systems, including genetic and pharmacologic perturbation of epigenetic regulators, together with longitudinal human studies demonstrating persistence of disease-associated chromatin states after clinical resolution. However, these data remain insufficient to establish causality for most epigenetic changes observed in human autoimmune skin disease.

An additional unresolved question is the relative contribution of tissue-resident versus recirculating memory T-cell populations to long-term disease persistence and recurrence. Current evidence most strongly supports a central role for TRM cells in mediating localized relapse, particularly in psoriasis, whereas circulating skin-homing memory populations likely contribute to systemic immune surveillance and replenishment of inflamed tissue. However, the relative importance of these populations remains incompletely defined because few human studies simultaneously profile resident and circulating immune compartments at single-cell resolution. Addressing this question will require integrated longitudinal analyses combining paired blood and skin sampling with single-cell, spatial, and functional epigenomic approaches.

### 9.4 Barriers to clinical translation of epigenetic therapies

Translating epigenetic insights into clinical practice presents several challenges. A central limitation is the lack of cell-type specificity of current epigenetic therapies. Agents targeting histone deacetylases, DNA methyltransferases, BET proteins, or EZH2 affect broad transcriptional programs across multiple cell types, raising concerns about off-target effects and systemic toxicity. While such risks may be acceptable in oncology, they are less tolerable in chronic inflammatory diseases requiring long-term treatment. Delivery also remains a major barrier. Effective epigenetic therapy in dermatology will likely require localized or skin-restricted approaches to minimize systemic exposure while targeting relevant cellular compartments. Early studies with topical histone deacetylase inhibitors provide proof of concept, but comparable strategies for other epigenetic targets remain underdeveloped. Another challenge lies in patient stratification. Epigenetic changes are heterogeneous across individuals and diseases, and it is unlikely that a single therapeutic approach will be universally effective. Biomarker-driven strategies will likely be essential to identify patients with specific epigenetic signatures who are most likely to benefit from targeted interventions. Finally, regulatory and safety considerations must be addressed, particularly for emerging approaches such as epigenome editing. Ensuring durability of effect without unintended long-term consequences will be critical for clinical translation.

### 9.5 Toward epigenetic reprogramming as a therapeutic strategy

Future research should prioritize longitudinal, multi-omic studies that integrate chromatin accessibility, DNA methylation, transcriptional activity, and spatial organization within the same tissue samples. Such approaches will be important for defining the temporal evolution of epigenetic states and identifying key regulatory pathways associated with disease persistence. Where feasible, these studies should incorporate paired pre- and post-treatment biopsies, clinically resolved lesions, serial sampling, and recurrent lesions from the same anatomical site, as these longitudinal designs provide the strongest evidence for persistent epigenetic memory and help distinguish durable epigenetic reprogramming from transient inflammatory responses. Advances in spatial and single-cell epigenomics are likely to play a central role in this effort by enabling mapping of regulatory programs within defined tissue niches. Integration of these datasets with clinical phenotypes and treatment responses will facilitate the development of predictive biomarkers and personalized therapeutic strategies. In the near term, the most clinically feasible translational applications are likely to include epigenetic biomarkers for patient stratification, treatment monitoring, and prediction of disease recurrence, together with localized or topical chromatin-modifying therapies that minimize systemic exposure. Longer-term goals include cell-selective epigenetic therapies and locus-specific epigenome editing, although these approaches will require substantial advances in targeted delivery, cellular specificity, long-term safety, and regulatory validation before routine clinical application. From a therapeutic perspective, the most promising direction lies in combination approaches that pair conventional immunomodulatory therapies with epigenetic interventions. While cytokine-targeted agents can rapidly suppress active inflammation, epigenetic therapies may offer the potential to destabilize pathogenic memory and improve long-term disease control. Finally, emerging technologies such as epigenome editing hold the potential to directly reprogram disease-associated regulatory elements in a locus-specific manner. Although still in early stages, these approaches represent a conceptual shift from broad immunosuppression to precise modulation of gene-regulatory circuits.

### 9.6 Limitations

The strength of evidence varies considerably across immune-mediated and autoimmune skin diseases. Psoriasis currently possesses the most comprehensive human epigenomic dataset, incorporating lesional skin methylomics, chromatin accessibility profiling, histone modifications, and single-cell analyses. In contrast, much of the evidence in atopic dermatitis, cutaneous lupus erythematosus, vitiligo, and alopecia areata remains derived from combinations of human lesional tissue, peripheral blood analyses, and mechanistic preclinical studies. Consequently, mechanistic conclusions should be interpreted within the context of the underlying evidence source, particularly when extrapolating findings from animal or *in vitro* systems to human disease. Furthermore, many studies rely on bulk tissue analyses, which cannot distinguish true cell-intrinsic epigenetic reprogramming from changes in cellular composition resulting from immune-cell infiltration or tissue remodeling. As a result, observed molecular signatures may partially reflect shifts in the proportions of immune and stromal cell populations rather than persistent epigenetic programs within specific cell types. Studies employing sorted cell populations, single-cell transcriptomic or epigenomic approaches, and paired longitudinal sampling before and after treatment provide stronger support for persistent immune memory by enabling the identification of stable, cell-specific molecular programs independent of tissue composition. Interpretation of existing studies is further complicated by potential confounding from treatment effects, anatomical biopsy site variation, disease duration and severity, and patient comorbidities, all of which may independently influence epigenetic and transcriptional profiles and are not uniformly controlled across studies. Additional limitations include the relatively small sample sizes of many epigenomic studies, limited independent replication across patient cohorts, and the scarcity of functional perturbation studies needed to establish causal relationships between specific epigenetic alterations and disease persistence.

## 10 Conclusion

Immune-mediated inflammatory and autoimmune skin diseases are increasingly recognized as disorders of immune dysregulation in which maladaptive epigenetic memory provides an important mechanistic framework for understanding disease persistence. By integrating genetic susceptibility with environmental exposure, epigenetic mechanisms may help explain disease chronicity, site specificity, and recurrence beyond traditional cytokine-centered models. Advances in single-cell, spatial, and multi-omic technologies are enabling increasingly detailed characterization of these regulatory programs, while emerging therapeutic strategies, including chromatin-targeting agents and epigenome editing, offer the potential to move beyond transient suppression toward modulation of pathogenic epigenetic programs. However, direct causal evidence in human disease remains limited, and the clinical utility of epigenetic biomarkers and targeted therapies requires validation in larger, longitudinal, and functional studies. Realizing this potential will require overcoming challenges in specificity, delivery, and patient stratification. As these technologies continue to evolve, maladaptive epigenetic memory may provide a valuable conceptual framework for understanding disease recurrence and identifying future therapeutic opportunities.
